# Computer assisted verbal autopsy: comparing large language models to physicians for assigning causes to 6939 deaths in Sierra Leone from 2019–2022

**DOI:** 10.1186/s12916-025-04584-z

**Published:** 2025-12-24

**Authors:** Richard Wen, Anteneh Tesfaye Assalif, Andy Sze-Heng Lee, Rajeev Kamadod, Asha Behdinan, Ronald Carshon-Marsh, Catherine Meh, Thomas Kai Sze Ng, Patrick Brown, Prabhat Jha, Rashid Ansumana

**Affiliations:** 1 Centre for Global Health Research, St. Michael’s Hospital, Unity Health Toronto and University of Toronto, 30 Bond St, Toronto, Ontario M5B 1W8 Canada; 2 College of Medical Sciences, Njala University, Bo, Sierra Leone

**Keywords:** Cause of death, Physicians, Computer-assisted diagnosis, Artificial intelligence, Natural language processing, Machine learning, Mortality, Surveillance, Mathematical models, Global health

## Abstract

**Background:**

Verbal autopsies (VAs) collect information on deaths in low and middle-income countries occurring outside healthcare facilities to estimate causes of death (CODs) for use in epidemiological or planning studies. Physician coding of VAs focused on the narrative of deaths and past symptoms is current best practice. Large language models (LLM) such as GPT-5 enable possible use of the narrative portion of VAs to assign CODs. However, there are few if any robust comparisons of LLMs to physician coding.

**Methods:**

We analyzed 6,939 VA records from a random sample of deaths in Sierra Leone (2019–2022) to compare five models: three LLMs (GPT-3.5, GPT-4, GPT-5) and two based on symptom algorithms (InterVA-5, InSilicoVA), against physician-assigned CODs. GPT models used narratives, whereas InterVA-5 and InSilicoVA relied on questionnaires. CODs were grouped into 19, 10, and 7 categories for adult, child, and neonatal deaths. We used cause specific mortality fraction (CSMF) accuracy and partial chance corrected concordance (PCCC) to assess population and individual-level agreement respectively, compared to the standard of physician coding. We stratified analyses by age group as CODs vary among neonates, children and adults.

**Results:**

Overall, GPT-5 outperformed all models (PCCC = 0.71), followed by GPT-4 (0.61), GPT-3.5 (0.56), InSilicoVA (0.44), and InterVA-5 (0.44). GPT-5 achieved the highest performance for adult (0.68), child (0.71), and neonatal (0.65) deaths. Across ages, performance increased from 1 month to 14 years and declined from 15 to 69 years. GPT-5, GPT-4, GPT-3.5, and InSilicoVA achieved the highest PCCC in 14, 7, 7, and 2 of the 30 CODs, respectively. At the population level, GPT-5 achieved the highest CSMF accuracy (0.9), while all other models had comparable performance (0.74–0.79).

**Conclusions:**

GPT models and InSilicoVA showed greater performance for specific CODs at the individual-level. GPT models demonstrated improvements over InterVA-5 and InSilicoVA models. This study provides foundational evidence for integrating LLM and algorithmic models with physician coding to improve the quality of VA data.

**Supplementary Information:**

The online version contains supplementary material available at 10.1186/s12916-025-04584-z.

## Background

Reliable mortality counts and accurate Cause of Death (COD) data are essential for guiding public health policy and reducing premature mortality [1–4]. However, civil registration and vital statistics systems remain incomplete in many low-income countries. Fewer than half of all deaths are registered, and among these, only 8% have an assigned COD [5]. To address this gap, Verbal Autopsy (VA) has been deployed as a scalable method for collecting mortality data and assigning likely CODs, particularly for deaths that occur outside of healthcare facilities, which account for more than half of all deaths [6–9].

VA involves two major components: survey and COD assignment [10–12]. In the survey component, trained interviewers use structured questionnaires and open narrative prompts to gather data from relatives or close contacts of the deceased. In the COD assignment component, physicians review these data to determine the most likely COD. However, reliance on physician assignment has been criticized for limited reproducibility and subjectivity [13–17]. To overcome these limitations, automated Computer Coded Verbal Autopsy (CCVA) methods such as InterVA [18] and InSilicoVA [15] have been developed and used in many VA studies. These models offer scalable and reproducible alternatives and have demonstrated comparable performance to physicians at the population level. However, their performance at the individual-level remains limited [19–23], while their reliance on structured questionnaire data often omits open narrative text, which can contain additional contextual and chronological information that may improve diagnostic accuracy [24–26]. A randomized trial evaluating dual physician coding with current CCVA methods concluded that physician coding was superior [19].

Recent advances in large language models (LLMs), trained on vast textual datasets using deep learning methods, have significantly improved natural language processing (NLP) capabilities. These include tasks such as question answering, code generation, and medical reasoning based on free text [27–30]. ChatGPT, developed by OpenAI and released in 2022, is a widely accessible LLM capable of generating human-like responses to natural language queries. Earlier versions (GPT-1 to GPT-3) scaled from 117 million to 175 billion parameters and were trained on data ranging from 5 GB to 45 TB [31]. In 2023, ChatGPT-4 was introduced, achieving human-level performance on a range of academic and professional benchmarks [32]. More recently, GPT-5, introduced in 2025, has been reported to demonstrate enhanced reasoning capabilities and reduced tendency to provide inaccurate or misleading information [33]. Given the underutilization of narrative free text in VA analysis and the capabilities of LLMs in processing such data, we conducted a study using VA records from Sierra Leone (2019–2022) to compare five models: three LLMs (GPT-3.5, GPT-4, and GPT-5) and two algorithms based on symptoms (InterVA-5 and InSilicoVA), against physician-assigned CODs. This work aims to evaluate the potential of LLMs in enhancing COD assignment from narrative data in low-resource settings.

## Methods

**Fig. 1:**
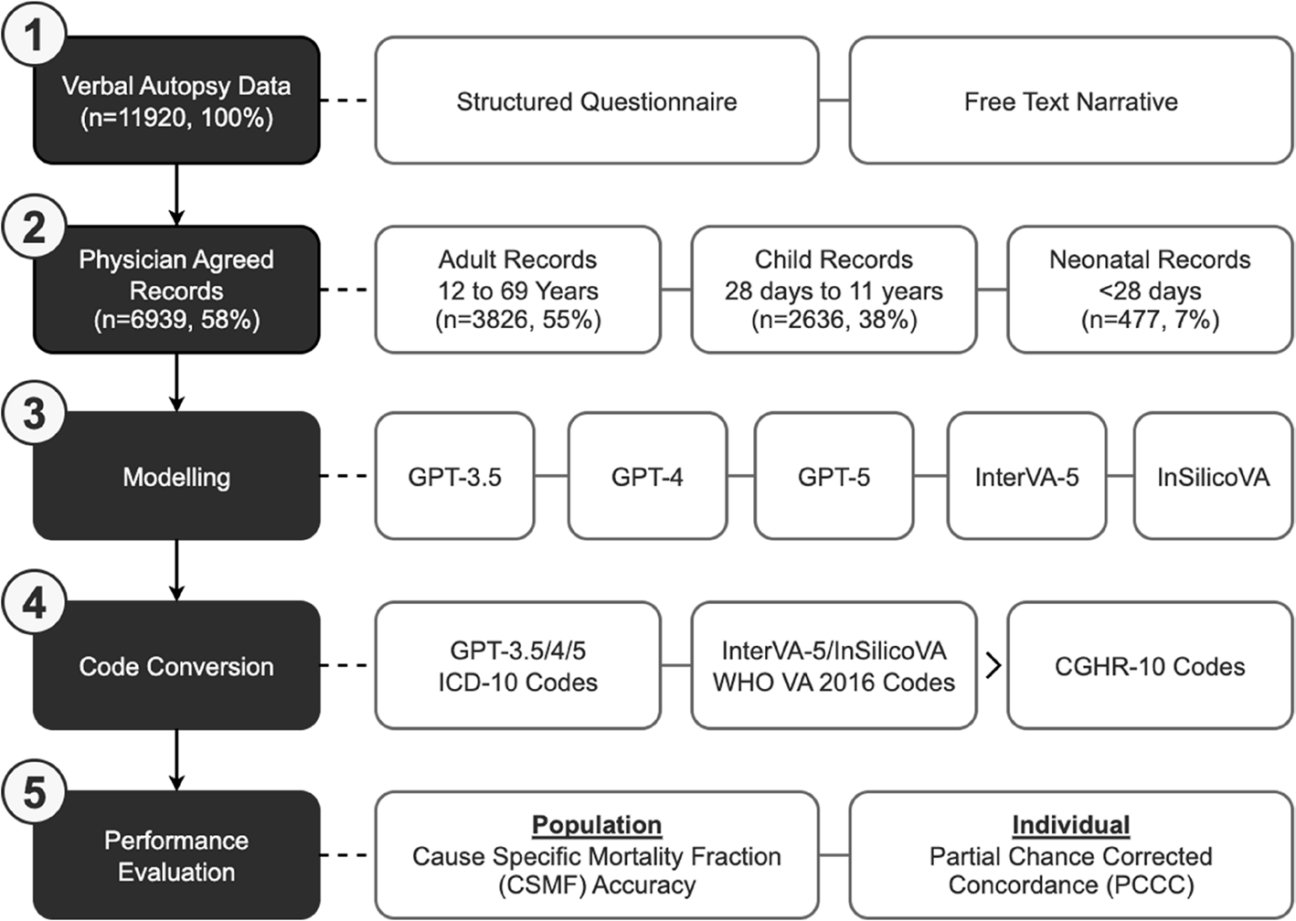
Flow diagram for verbal autopsy coding comparison of 6939 sample deaths in Sierra Leone. Verbal autopsy data containing 11,920 sample deaths were initially collected from in-field surveys, and filtered to 6939 records where two randomly assigned physicians agreed on the cause of death. Five computer models GPT-3.5, GPT-4, GPT-5, InterVA-5, and InSilicoVA were compared to physicians using standardized CGHR-10 codes, and evaluated using individual PCCC and population CSMF accuracy metrics

This section outlines the methodology used to compare cause of death (COD) assignments from five models, GPT-3.5, GPT-4, GPT-5, InterVA-5, and InSilicoVA, with physician-determined CODs, as summarized in Fig. 1. The dataset was first filtered to include only records with physician agreement, as described in Sect. 2.1. Section 2.2 details the input formats and output structures of the five models. Section 2.3 presents the evaluation framework, which compares model outputs to physician assigned CODs using both population-level and individual-level performance metrics. Additional details are provided in Appendix B.

### Verbal Autopsy (VA) data

A total of 11,920 verbal autopsy (VA) records were obtained from the HEAL-SL study, a nationally representative survey covering about 5% of the homes in Sierra Leone, and collected from about 660 small sampling units [34, 35]. HEAL-SL employs dual-coded Electronic Verbal Autopsy (EVA). Each record was independently reviewed by two randomly selected physicians, who assigned COD codes based on the International Classification of Diseases, 10th Revision (ICD-10) [36]. Agreement between physician-assigned CODs was evaluated using Central Medical Evaluation Agreement 10 (CMEA-10) codes, which group related ICD-10 codes into broader, clinically similar categories [37] (see Additional File 1). If both codes fell within the same CMEA-10 group, the record was considered in agreement. Disagreements entered a reconciliation phase, where each physician (blinded to the other physician’s identity) was shown both the assigned codes and the reasoning from the other physician. Physicians could then (1) retain their original code, (2) adopt the other physician’s code, or (3) assign a new code. Records that remained unresolved proceeded to adjudication, where a senior physician reviewed all reasoning and assignments and issued a final COD.

To ensure comparability with physician coding, only records with initial physician agreement were used in this study, as such cases provide higher confidence in the COD assignment [16, 38, 39]. From the original dataset, 6,942 records met this criterion. All ICD-10 codes were then standardized to CGHR-10 categories (see Appendix A), which group causes into 19, 10, and 7 categories for adults (12–69 years), children (28 days to 11 years), and neonates (under 28 days), respectively. After excluding three records without a valid CGHR-10 category, a total of 6,939 physician-agreed records (3,826 adult, 2,636 child, and 477 neonatal) were used for model comparison and performance evaluation. Further details on data preprocessing are provided in Appendix B.1, with COD and age group distributions summarized in Tables B4 and B5.

### Modelling

Five computational models were used to assign causes of death (CODs) for each of the 6,939 physician-agreed verbal autopsy (VA) records: GPT-5, GPT-3.5, GPT-4, InterVA-5, and InSilicoVA. InterVA-5 and InSilicoVA are widely used statistical models within the OpenVA framework for COD assignment in VAs [11, 19, 20, 22, 23, 40–42]. InterVA-5 applies a Bayesian probabilistic approach, using a standardized set of symptoms and expert-derived conditional probabilities to assign the most likely COD based on maximum probability [18, 43, 44]. InSilicoVA extends this approach by incorporating a hierarchical Bayesian framework and Markov Chain Monte Carlo methods [45–47], allowing for quantification of uncertainty, individual-level probability estimates, and the integration of additional data sources [15]. GPT-3.5 [48], GPT-4 [32], and GPT-5 [33] are large language models (LLMs) that generate human-like text by learning from large amounts of textual data [49]. These models are trained using reinforcement learning from human feedback [50–53], enabling them to follow natural language instructions while generating human-level responses. GPT-4 demonstrated improvements over GPT-3.5, including more recent training data, enhanced reasoning capabilities, and multimodal input–output functionality (e.g., text, image, voice) [31], while GPT-5 has been reported in preliminary studies to outperform GPT-4 across multiple evaluation domains with enhanced reasoning capabilities and reduced hallucination [54–56].

For GPT-3.5, GPT-4, and GPT-5, the following user prompt was used to instruct each model to produce COD assignments as ICD-10 codes (mirroring physician practice), where < *age* > and < *sex* > from the questionnaire, and < *narrative* > from the narratives, were replaced with values from the data:

*Determine the underlying cause of death and provide the most probable ICD−10 code for a verbal autopsy narrative of a* <*age*> *years old* <*sex*> *death in Sierra Leone:* <*narrative*>

InterVA-5 and InSilicoVA used structured questionnaire data, which were converted into OpenVA-compatible format [42]. Both models produced COD assignments coded using the WHO 2016 VA standard [57]. The narratives consist of unstructured text describing each death, including reported events, circumstances, and symptoms, recorded by trained verbal autopsy (VA) surveyors through interviews with family, next-of-kin, or caregivers of the deceased. In contrast, the questionnaire contains structured questions for gathering standardized information from interviewees that aid in determining the COD. Narratives provide additional contextual and temporal information (e.g., event circumstances, symptom chronology, social factors, health behaviours, semantics) that may improve diagnostic accuracy, often absent from questionnaires [24–26]. Thus, the narratives and questionnaire do not contain equivalent information and differ in both content and structure, with GPT models leveraging free-text narratives and InterVA-5/InSilicoVA relying primarily on questionnaire responses. To ensure comparability across models, all output CODs were mapped to the CGHR-10 classification system for evaluation relative to physician-assigned CODs. Parameters for InterVA-5 and InSilicoVA were set to reflect disease assumptions of Sierra Leone, while parameters for GPT-3.5/4/5 were set to produce more deterministic outputs. Further details on model input formats, output mappings, and code conversion procedures are provided in Appendix B.2.

### Performance evaluation

Model performance was assessed at both the population and individual levels by comparing each model’s CGHR-10 COD assignments to those of physicians for all 6,939 records. Cause-specific mortality fraction (CSMF) accuracy was used to evaluate agreement at the population level adjusting for maximum total error from the worst model (see Appendix B.3.1), while partial chance corrected concordance (PCCC) was used to assess individual-level agreement adjusting for random chance (see Appendix B.3.2) [58]. Both metrics range from 0 to 1, where higher values indicate stronger similarity with physician assignment. Given that model performance can vary by age and different CODs [40, 41, 59], both CSMF accuracy and PCCC were calculated overall and stratified by age group (adult, child, neonatal), CGHR-10 COD, and age at death. For adult and child groups, metrics were computed in five-year age bands for records with age at death of one year or older, and five-month bands for records between 28 days and one year. For the neonatal group, evaluations were conducted separately for age intervals of 0–6 days and 7–27 days. PCCC and CSMF accuracy have both been well-studied and widely used to report performances for CCVA models [19–21, 24, 60, 61]. Additional details on the evaluation strategy and metric calculations are provided in Appendix B.3.

## Results

### Overall performance

**Fig. 2:**
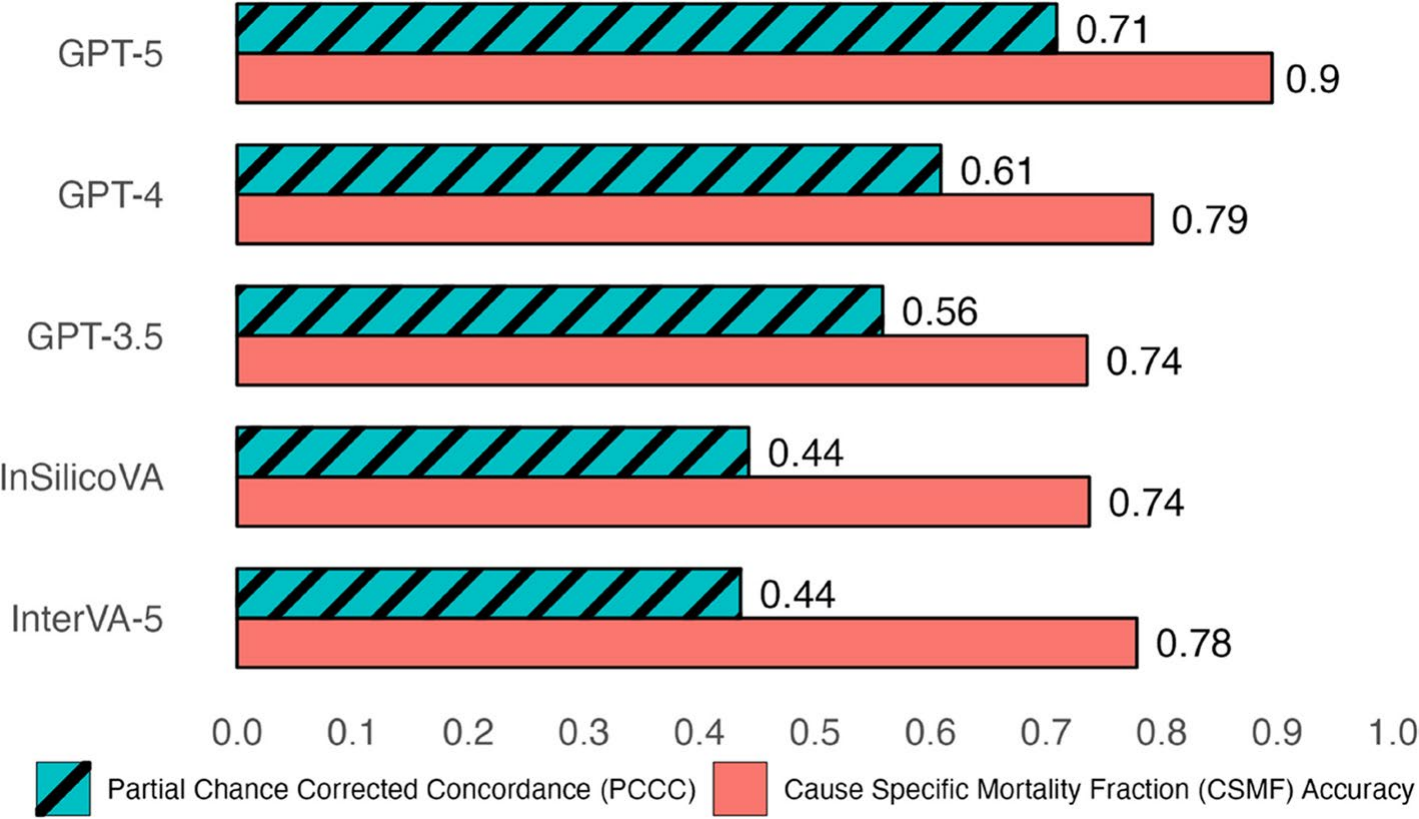
Individual (PCCC) and population (CSMF accuracy) level verbal autopsy coding performance of all 6939 deaths. PCCC and CSMF accuracy values range from 0 to 1. PCCC values of 1 indicate complete agreement with physician coding per individual death, while CSMF accuracy values of 1 indicate complete agreement with physician coding per cause, irrespective of individual deaths

**Fig. 3:**
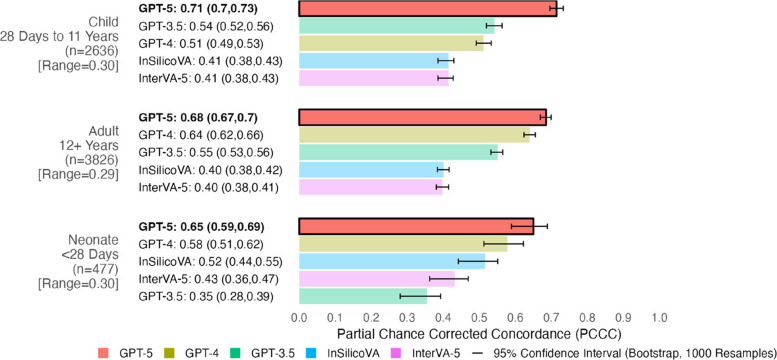
Individual-level verbal autopsy coding performance by age group. PCCC values range from 0 to 1, with 1 indicating complete agreement with physician coding per individual death. Range represents the difference between the maximum and minimum PCCC values across all models per age group. 95% confidence intervals for PCCC were computed using bootstrapping (1000 resamples with replacement). Age groups are sorted by their model with the highest PCCC (highlighted in bold and opaque bars at the top of each age group), while models are sorted from highest to lowest PCCC within each age group

**Fig. 4:**
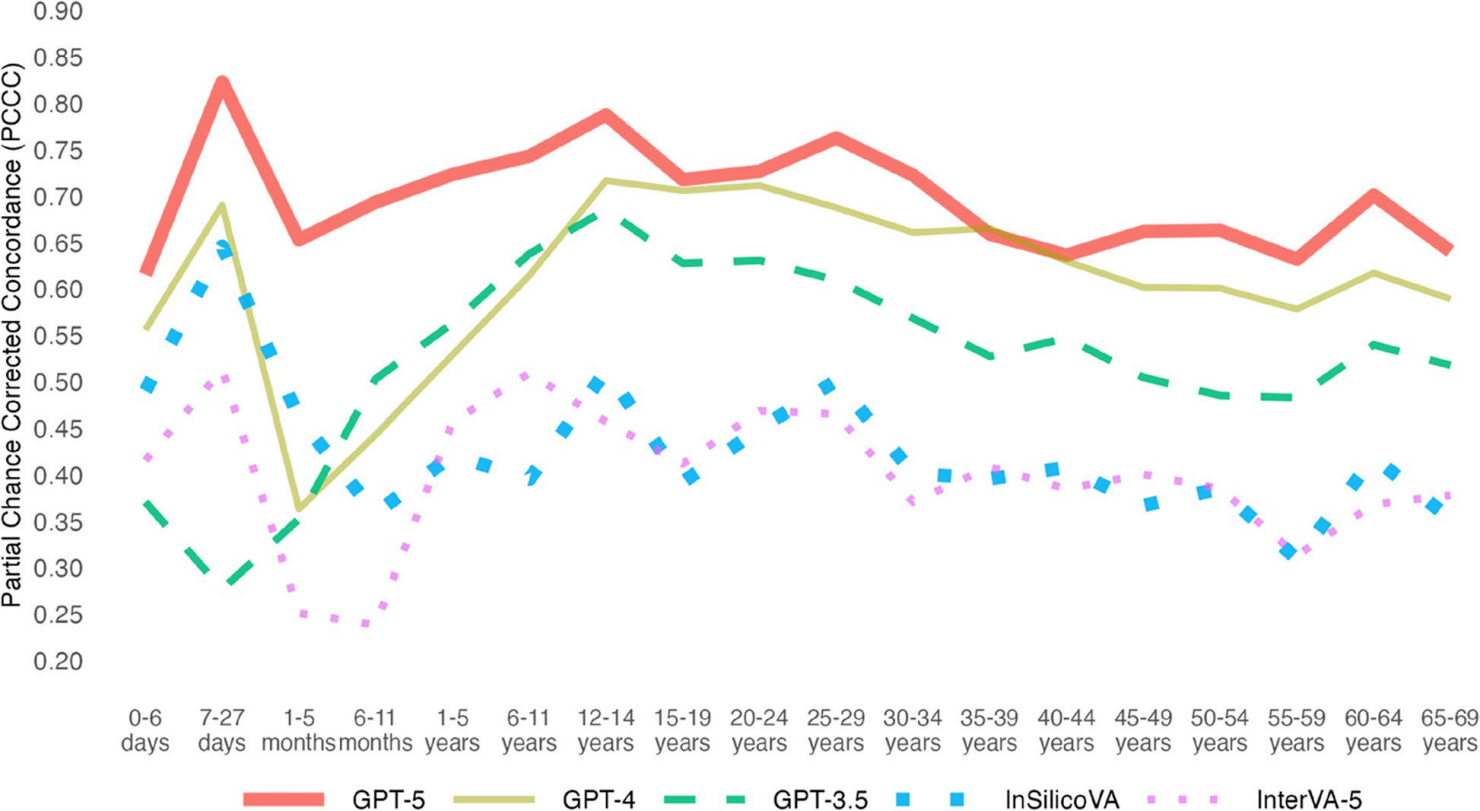
Individual-level model performance by age of deceased. PCCC values range from 0 to 1, with 1 indicating complete agreement with physician coding per individual death. Ages 0–27 days represent neonatal deaths, ages 1–11 months represent child deaths, and ages 12–69 years represent adult deaths

At the population level, GPT-5 achieved the highest performance (0.9 CSMF accuracy), while all other models had similar performances (0.74–0.79 CSMF accuracy). The remaining analyses focus on individual-level performances measured by PCCC. GPT-5 demonstrated the highest performance (0.71) followed by GPT-4 (0.61), GPT-3.5 (0.56), InSilicoVA (0.44), and InterVA-5 (0.44) (Fig. 2). GPT-3.5/4/5 had improvements from 0.12–0.27 in the PCCC over InSilicoVA and InterVA-5, while GPT-5 improved over GPT-4 (+ 0.1 in the PCCC) and GPT-4 slightly improved over GPT-3.5 (+ 0.05 in the PCCC). Figure 3 shows the individual performance across three age groups (adult, child, and neonate). GPT-5 had the best performance for all age groups (0.68, 0.71, and 0.65 for adult, child, and neonatal records respectively). InSilicoVA and InterVA-5 performed the worst for adult and child records (less than 0.5), while GPT-3.5 performed the worse for neonatal records (0.35). Performance varied from 0.29–0.3 in the PCCC across the three age groups. Across ages, a trend in performance was observed for all models (Fig. 4). In adults, performance decreased with age for 12 to 59 years (from ~ 0.75 down to ~ 0.35), suggesting greater difficulty in assigning CODs among older adults, with a modest improvement observed after age 59. Among children and neonates, performance improved from 5 months to 11 years (from ~ 0.25 towards ~ 0.75), indicating greater model reliability as developmental age advanced. The highest and lowest performances were observed for ages 12–29 years (0.4–0.75) and 1–11 months (0.25–0.68) respectively. In terms of individual performance by COD, GPT-3.5/4/5 consistently outperformed InterVA-5 and InSilicoVA for most leading CODs identified in prior Sierra Leone studies [35, 62] as seen in Table 1. A majority of CODs had at least one high performing model (0.78–0.99.78.99 for 21 of 30 CODs). More details are available in Sections 3.2–3.4.2.4.

**Table 1:** Top ten leading causes of death for Sierra Leone in 2023 and best performing models for verbal autopsy coding

Top 10 Leading Cause of Death (∼71% of ∼76 K deaths)^a^	Deaths (% of 76 K)^b^	Best Model(s) at the individual-level
Malaria	16,075 (21%)	GPT-5
Other Infections	11,777 (16%)	GPT-3.5/GPT-5/InSilicoVA
Ischaemic heart and other vascular	5,747 (8%)	GPT-5
Diarrhoea	4,285 (6%)	GPT-4/GPT-5
Stroke	4,262 (6%)	GPT-5
Pneumonia	3,074 (4%)	GPT-4/InSilicoVA
Birth asphyxia and birth trauma	2,431 (3%)	GPT-5
Tuberculosis	2,399 (3%)	GPT-5
Low birth weight/preterm	1,570 (2%)	GPT-5
Asthma and chronic respiratory	1,551 (2%)	GPT-3.5

^a^Other infections and severe systemic/localized infections were generalized into infections. Appendix, hernia, intestinal and Peptic ulcer/gastroesophageal causes did not have comparable CGHR-10 codes and were omitted from the top ten

^b^Percentage of ∼76 thousand (K) total deaths [62]. Numbers are rounded

### Performance by COD for 3826 adult records (12 to 69 years)

**Fig. 5:**
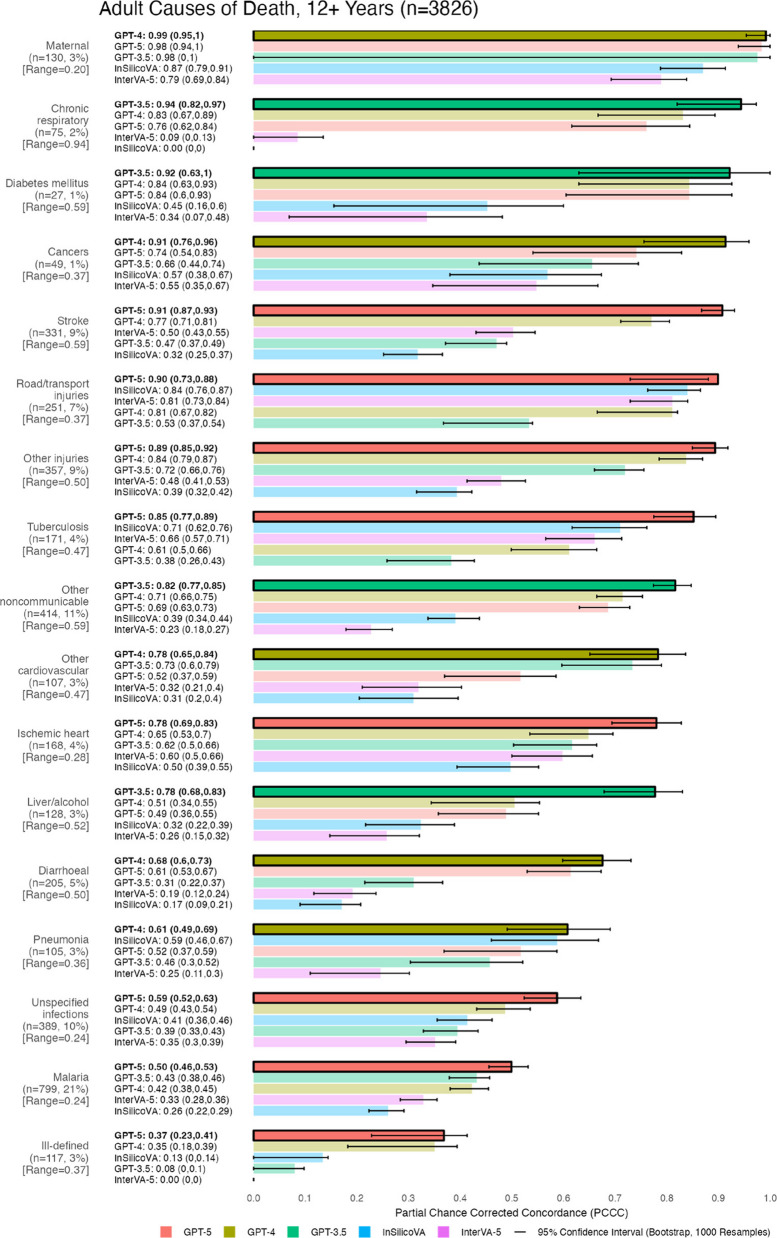
Individual-level model performance for adult causes of death. PCCC values range from 0 to 1, with 1 indicating complete agreement with physician coding per individual death. Range represents the difference between the maximum and minimum PCCC values across all models per cause of death. 95% confidence intervals for PCCC were computed using bootstrapping (1000 resamples with replacement). Model names are followed by their PCCC and confidence intervals in brackets. Causes of death are sorted by their model with the highest PCCC (highlighted in bold text and opaque bars at the top of each cause of death), while models are sorted from highest to lowest PCCC within each cause of death. Suicide (*n* = 3, < 1%) was excluded due to low sample size

Figure 5 presents performance across 17 adult CODs. At least one model performed well (0.78–0.99) for 12 of the 17 CODs. GPT-5 achieved the highest individual-level performance for 8 of 17 CODs (0.36–0.91), followed by GPT-4 for 5 CODs (0.61–0.99), and GPT-3.5 for 4 CODs (0.78–0.94). InterVA-5 had the lowest performance for 9 CODs (0–0.79), InSilicoVA for 6 CODs (0–0.5), and GPT-3.5 for 2 CODs (0.38 and 0.53). The greatest improvements of GPT-3.5/4/5 over InSilicoVA and InterVA-5 were observed in chronic respiratory diseases (+ 0.74–0.94 in the PCCC), while the smallest improvements were for ischemic heart disease (+ 0.02–0.28 in the PCCC). All models performed well for maternal conditions (0.79–0.99), but poorly for unspecified infections (0.35–0.59), malaria (0.26–0.5), and ill-defined CODs (0–0.37). GPT-5 showed performance improvements over all other models for stroke (+ 0.14–0.59 in the PCCC) and tuberculosis (+ 0.14–0.47 in the PCCC). GPT-4 demonstrated similar gains for cancers (+ 0.17–0.36 in the PCCC), while GPT-3.5 improved over other models for chronic respiratory diseases (+ 0.1–0.94 in the PCCC), diabetes (+ 0.08–0.58 in the PCCC), other non-communicable diseases (+ 0.11–0.59 in the PCCC), and liver and alcohol-related diseases (+ 0.25–0.52 in the PCCC). Performance variability across models was most pronounced for chronic respiratory diseases (range: 0.94), while narrower differences were observed for maternal conditions (range: 0.20), malaria (range: 0.24), ischemic heart disease (range: 0.28), and unspecified infections (range: 0.24). CODs with high physician agreement and models that performed well include road/transport injuries (0.81–0.9 for four models, 75% agreement), and other injuries (0.72–0.89 for three models, 72% agreement), while models performed poorly for malaria despite high agreement (0.26–0.5 PCCC, 77% agreement). CODs with low physician agreement and models that performed poorly include pneumonia (0.25–0.61 for all models, 34% agreement) and unspecified infections (0.29–0.59 for all models, 37% agreement), while cancers (0.74–0.91 for two models, 20% agreement) had high performing models despite low physician agreement. For more details on physician agreement across adult CODs, see Table B4 in Appendix B.1.

## Performance by COD for 2636 child records (28 Days to 11 Years)

**Fig. 6:**
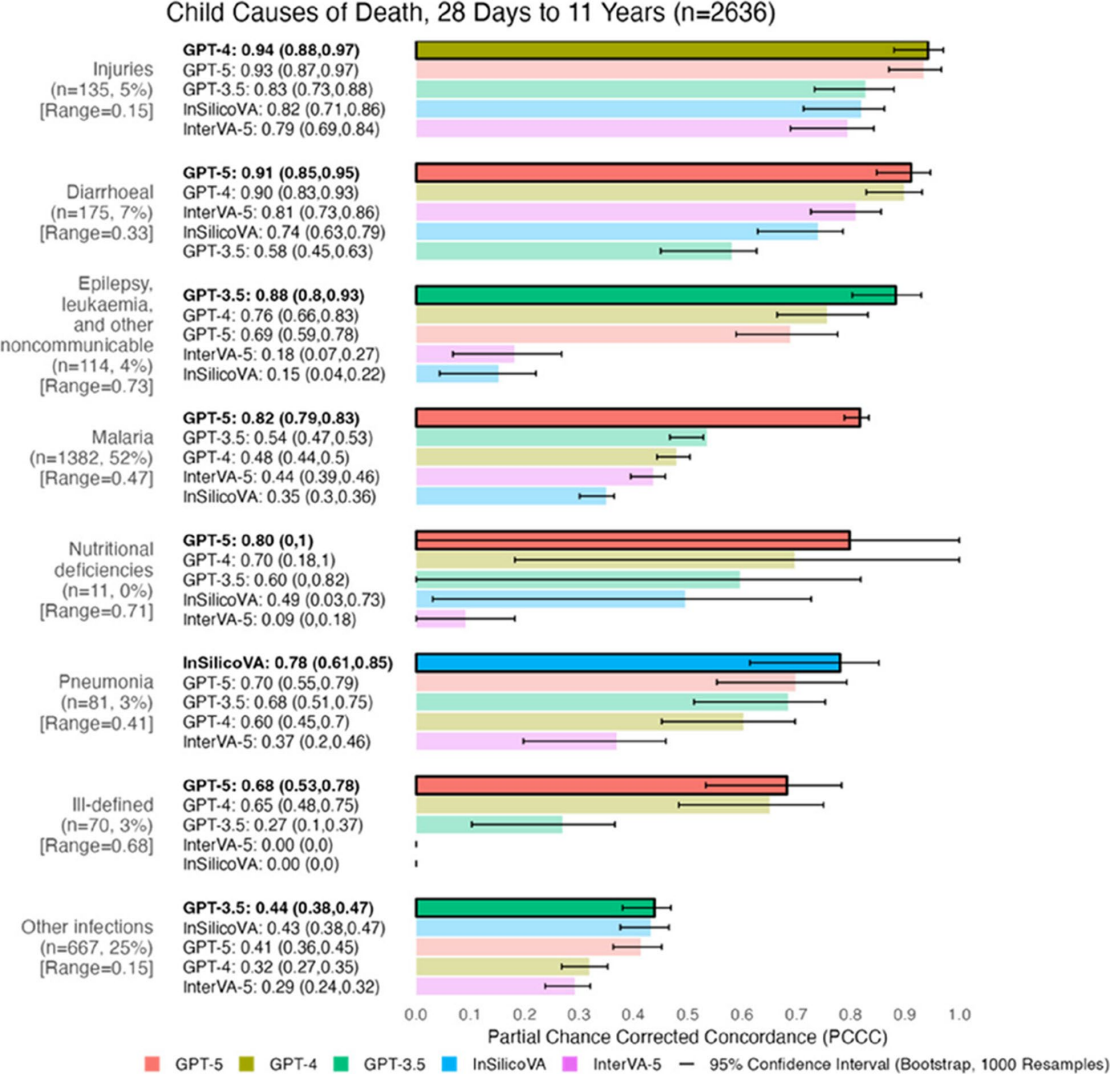
Individual-level model performance for child causes of death. PCCC values range from 0 to 1, with 1 indicating complete agreement with physician coding per individual death. Range represents the difference between the maximum and minimum PCCC values across all models per cause of death. 95% confidence intervals for PCCC were computed using bootstrapping (1000 resamples with replacement). Model names are followed by their PCCC and confidence intervals in brackets. Causes of death are sorted by their model with the highest PCCC (highlighted in bold text and opaque bars at the top of each cause of death), while models are sorted from highest to lowest PCCC within each cause of death. Congenital anomalies (*n* = 1, < 1%) was excluded due to low sample size

Figure 6 shows individual-level performance across 8 child CODs. Models performed well (0.78–0.94) for 6 of the 8 CODs. GPT-5 achieved the highest PCCC for 4 of the 8 CODs (0.68–0.91), followed by GPT-3.5 for 2 CODs (0.44 and 0.88), GPT-4 for one COD (0.94), and InSilicoVA for one COD (0.78). InterVA-5 had the lowest performance for 4 CODs (0.09–0.79), InSilicoVA for 3 CODs (0–0.35), and GPT-3.5 for one COD (0.58). All models performed well for injuries (0.79–0.94), while showing lower performance for other infections (0.29–0.44). GPT-5 demonstrated an improvement over other models for malaria (+ 0.28–0.47 in the PCCC), while demonstrating stronger performance along with GPT-4 for ill-defined CODs (+ 0.38–0.68 in the PCCC) versus all other models. Performance differences exceeding 0.60 in the PCCC were observed for epilepsy, leukaemia, other communicable diseases (range: 0.73), ill-defined causes (range: 0.68), and nutritional deficiencies (range: 0.71). In contrast, narrower differences (less than 0.30 in the PCCC) were seen for injuries (range: 0.15) and other infections (range: 0.15). CODs with high physician agreement and models that performed well include malaria (0.82 PCCC for GPT-5, 85% agreement) and injuries (0.79–0.94 for all models, 76% agreement). Nutritional deficiencies (0.7 and 0.8 for GPT-4 and GPT-5, 39% agreement) and pneumonia (0.7 and 0.78 for GPT-5 and InSilicoVA, 31% agreement) had high performing models despite poor agreement. For more details on physician agreement across child CODs, see Table B4 in Appendix B.1.

## Performance by COD for 477 neonatal records (Under 28 Days)

**Fig. 7:**
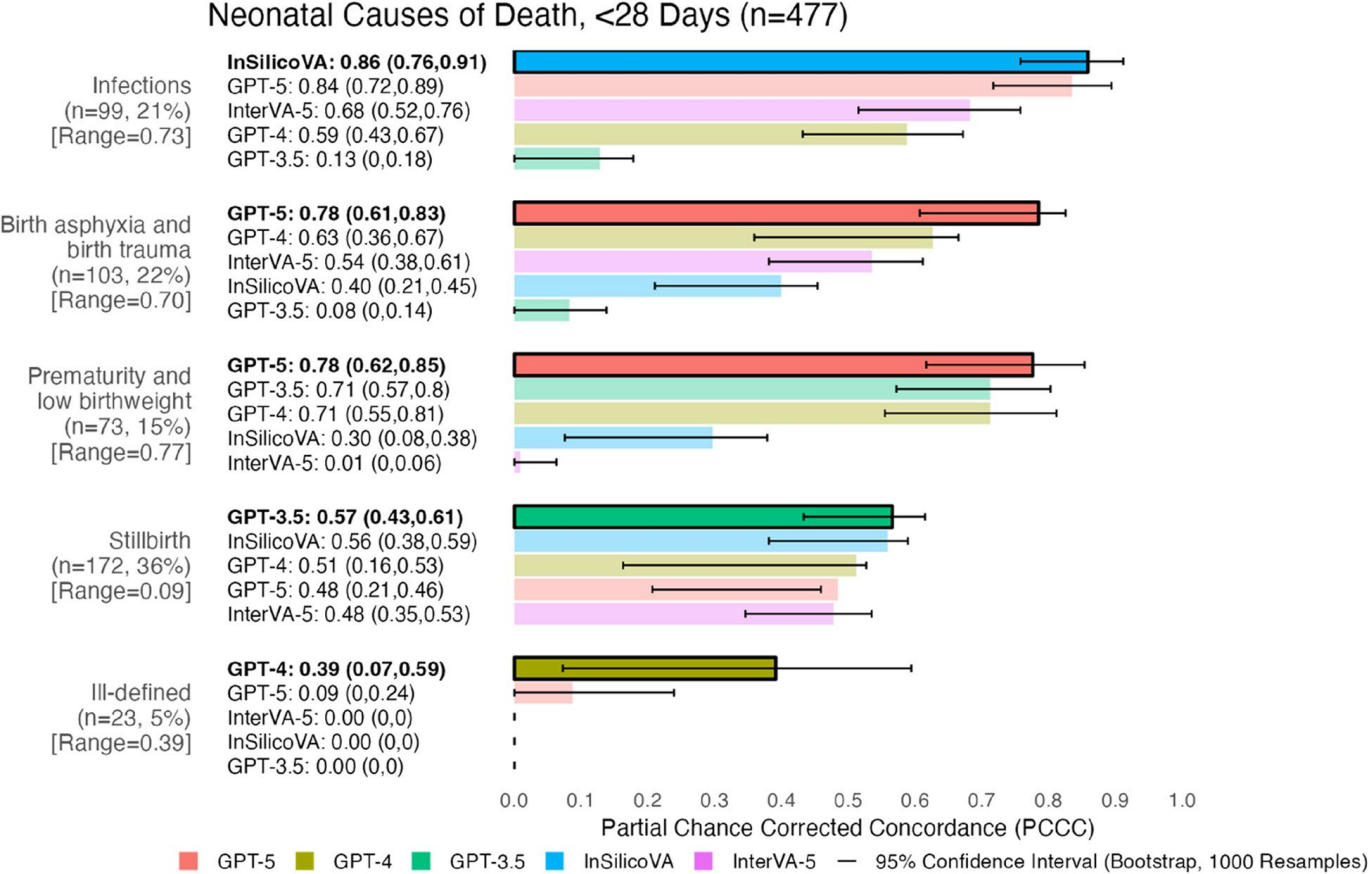
Individual-level model performance for neonatal causes of death. PCCC values range from 0 to 1, with 1 indicating complete agreement with physician coding per individual death. Range represents the difference between the maximum and minimum PCCC values across all models per cause of death. 95% confidence intervals for PCCC were computed using bootstrapping (1000 resamples with replacement). Model names are followed by their PCCC and confidence intervals in brackets. Causes of death are sorted by their model with the highest PCCC (highlighted in bold text and opaque bars at the top of each cause of death), while models are sorted from highest to lowest PCCC within each cause of death. Congenital anomalies (*n* = 2, < 1%) and other causes (*n* = 5, 1%) were excluded due to low sample size

Figure 7 shows model performance across 5 neonatal CODs. Models performed well (0.78–0.86) for 3 of the 5 CODs. GPT-5 achieved the highest performance for 2 of the 5 CODs (both 0.78), while InSilicoVA, GPT-3.5, and GPT-4 had the highest performance for one COD each (0.86, 0.57, and 0.39 respectively). GPT-3.5 showed the lowest performance for 3 CODs (0–0.13), and InterVA-5 for 2 CODs (0.01 and 0.48). Performance was similar across all models for stillbirths (0.48–0.57). Only GPT-5 and GPT-4 achieved a PCCC greater than zero for ill-defined deaths. InSilicoVA outperformed most other models for infections (0.86) with gains of + 0.18–0.73 in the PCCC, except for GPT-5, which performed very similarly (0.84). Larger performance differences between models were observed for infections (range: 0.73) and prematurity and low birthweight (0.77), while lower differences were seen in stillbirth (range: 0.09). Stillbirth (76% agreement) had low performing models (0.48–0.57 for all models) despite high physician agreement. For more details on physician agreement across neonatal CODs, see Table B4 in Appendix B.1.

## Discussion

As model performance varied by disease and age, the findings suggest models trained for each COD to maximize performance across causes, and validating that performance across ages align with expectations from clinical literature [63, 64]. From the results, we observed that GPT-3.5/4/5 consistently outperformed InterVA-5 and InSilicoVA for most CODs. A key advantage of GPT models was their ability to process and generate natural language text as input and output. Unlike InterVA-5 and InSilicoVA, GPT models assign CODs using the ICD-10 standard, mirroring physician practice. In contrast, InterVA-5 and InSilicoVA rely exclusively on structured WHO VA 2016 questionnaires and assign CODs using broader WHO VA 2016 codes. This dependency necessitates ongoing maintenance and conversion between questionnaire versions and coding systems, reducing interoperability and comparability across models. In addition, rarer diseases, underrepresented in questionnaire data, are better contextualized through external knowledge (e.g., web sources, journals, books) embedded in GPT models. Notably, GPT models were required to assign CODs from thousands of possible ICD-10 codes yet outperformed InterVA-5 and InSilicoVA, which operated on the comparatively fewer WHO VA codes, previously shown in the literature to reduce the likelihood of misclassification [21, 65–67]. The flexibility of GPT models in handling unstructured data allows them to capture latent and ambiguous information, such as health-seeking behaviors and social context, which are not encompassed by standardized VA codes or structured questionnaires [24, 26].

Although GPT models improved over InterVA-5 and InSilicoVA models, several limitations exist. Despite GPT-3.5/4/5 configured for more deterministic outputs (see Appendix B.2.2), a brief experiment in Appendix C revealed that GPT-3.5/4/5 did not assign consistent CODs when repeated on identical records. Thus, GPT models were inherently non-deterministic, as identical outputs across runs were not guaranteed [68–70]. Nonetheless, adjusting model parameters (e.g., temperature, seed, reasoning, verbosity) can improve output consistency, though complete stability is not ensured in practice [71–73]. In contrast, InterVA-5 and InSilicoVA provide assignments with probabilities for alternative causes, which was made feasible by calculating probabilities using repeated runs without costs. With the time and costs of running GPT models, repeated runs to evaluate output reliability in a robust manner was out of the scope of this study, where hundreds or thousands of repetitions substantially increase time and costs required by LLMs [74–76]. Recent studies have reported emerging token-based approaches to measuring and quantifying uncertainty, but have yet established best practices or standards for assessing the reliability of LLMs [77]. Another limitation common to all models was their reliance on past training data, limiting potential to detect new or emerging diseases (e.g., COVID-19). This is often remedied with re-training or updating models with new data or knowledge [78–80]. We also note that GPT-3.5/4/5 required data sent to external servers, raising significant privacy concerns from reliance on third-party services [81, 82]. In contrast, InterVA-5 and InSilicoVA are run on local systems under the control of the data owner. Privacy concerns are commonly addressed with the application of data anonymization techniques (e.g., generalization, suppression, de-identification) to remove or hide sensitive information [83–85]. While jurisdictions, such as the European Union, enforce strict protections under the general data protection regulation, most low‑ and middle‑income countries are only beginning to formalize regulatory frameworks for data protection and artificial intelligence governance [86–88]. As technology improves, larger GPT models may be possible on local systems, while currently, smaller LLMs exist as an alternative when adequate expertise and computing resources are available [89–91]. Although this study rigorously compares computer algorithms for COD assignment in Sierra Leone, the extent to which these findings are generalizable in other geographic or epidemiological contexts remains limited. Variations in local mortality profiles, linguistic expression, health system infrastructure, and culturally specific interpretations of illness shape the content and structure of VA narratives and questionnaires [92–94]. Similar to our results, we expect LLMs (GPT-3.5/4/5) to outperform statistical models (InterVA-5, InSilicoVA) overall and across most CODs, when applied to other regions. LLMs leverage sources of information available on the web (e.g., news, books, manuals, reports) containing knowledge for generalizing to other areas, such as widely known or changing regional variations in mortality and culture [80, 95–98]. In contrast, the information from these sources are often not up to date or captured by structured training data in statistical models. Nonetheless, sources leveraged by LLMs hold bias, and performance is not guaranteed when applied to regions underrepresented in the training data [98, 99]. Given ongoing efforts to scale and integrate VA systems for mortality surveillance across diverse low- and middle-income countries, further validation across more geographically representative VA datasets is essential to evaluate model robustness, adaptability, and operational utility in practice [100–102].

**Fig. 8:**
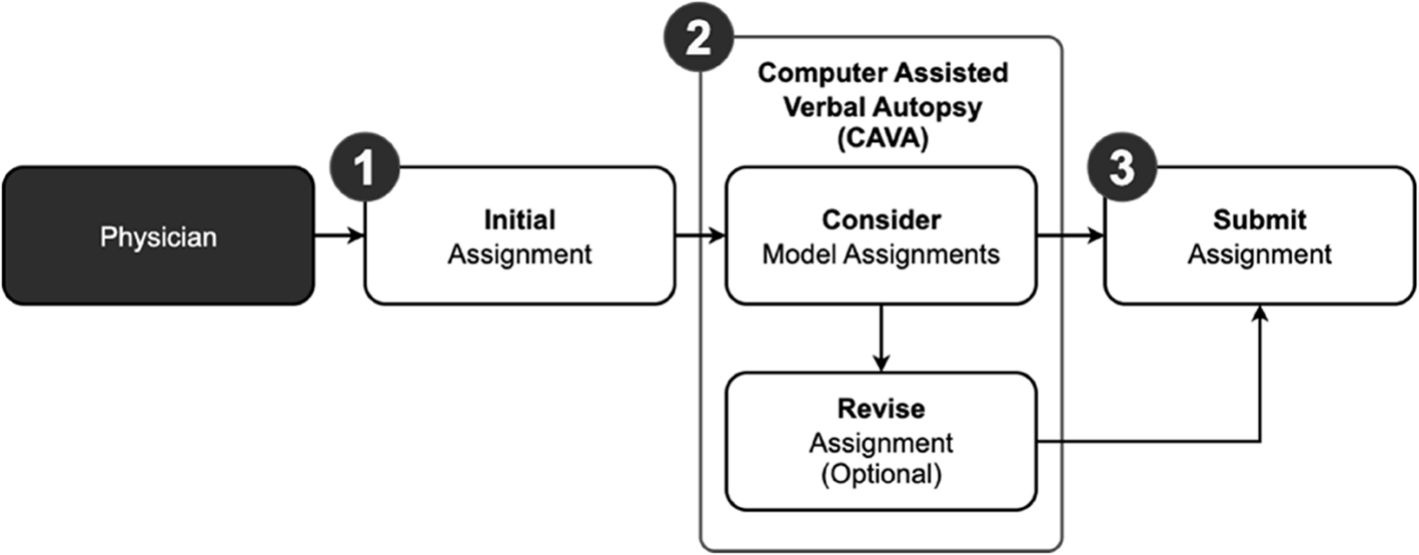
Computer Assisted Verbal Autopsy (CAVA) integrated into physician coding as a three-step process. The first step involves the physician assigning an initial cause of death to a record without considering causes of death provided by models. The second step is the addition of CAVA, where the physician can compare their initial assignment in step one to model assignments and optionally choose to revise their initial assignments. The third step submits the record with either the initial or revised assignment to the verbal autopsy coding system

This study establishes a basis for Computer Assisted Verbal Autopsy (CAVA), the integration of computer models into VA systems paired with physician assignment. Model-generated COD suggestions can be offered to physicians after their initial assignment, enabling reconsideration or confirmation of CODs as seen in step 2 of Fig. 8. We highlight that this study and earlier randomized evaluation of algorithms compared to the physician standard emphasize the core need to have physician coding of verbal autopsies [19–21]. The HEAL-SL method of drawing on physicians during their non-clinical time and using rigorous online methods with dual anonymous coding are scalable to other settings [38]. At the time of analysis, GPT-3.5 cost ~ $0.02 USD per 100 records, GPT-4 cost ~ $1.65 USD per 100 records, and GPT-5 cost ~ $0.05 USD per 100 records [103], while InterVA-5 and InSilicoVA were freely available as open-source software. These costs complement physician review, which is already affordable at ~ $1 USD per household (including field survey) in settings like India [13, 14]. The main costs of VA studies are in the field work, so the marginal costs of physician coding are small, and indeed our study suggests that quality enhancements introducing LLM and algorithms are very cost-effective. Given recent studies supporting improvement in physician diagnosis from LLM assistance [104, 105], we foresee the potential of alternative COD suggestions from computer models reducing physician disagreement and frequency of ill-defined records. Most CODs in our study have physician agreements between 50–60% with the lowest agreement at 20% and highest at 85% (Table B4), while the models evaluated performed well for most CODs (21 of 30 CODs). Hence, there is opportunity for models to further improve agreement amongst CODs. The HEAL-SL platform now incorporates LLM and algorithms, and the platform is available for use openly by other research and programmatic groups worldwide [34]. Presently, CAVA (using GPT-4, InterVA-5, and InSilicoVA) is integrated, with future work planned to evaluate the impact of CAVA on physician assignment.

## Conclusions

This study evaluated the performance of GPT-3.5, GPT-4, GPT-5, InterVA-5, and InSilicoVA models against physicians in assigning CODs for 6,939 VA records from Sierra Leone (2019–2022). At the individual-level, GPT models outperformed InSilicoVA/InterVA-5 by 0.14 to 0.27 PCCC with GPT-5 having the highest performance of 0.71 PCCC. By COD, GPT-5 performed best for 15 of the 30 CODs, while GPT-4, GPT-3.5, and InSilicoVA performed the best for 7, 7, and 2 CODs. Performance increased (∼0.25–0.75 PCCC) as children and neonates matured (0 days to 14 years) and decreased (∼0.7–0.35) with adult aging (15 to 69 years). At the population level, GPT-5 had the highest performance with a CSMF accuracy of 0.9, while all other models achieved similar performance (0.74–0.79). These findings suggest that combining models tailored to specific CODs and age groups may better optimize performance relative to physicians. All models demonstrated scalability and on-demand availability, enabling COD estimation and alternative diagnoses in low-resource or physician-scarce settings. The NLP capabilities of GPT models enabled performance closer to physician coding, but issues remain with reproducibility, reliance on historical data, data privacy, and verification in other regions. This study demonstrates the strengths of LLMs for COD assignment, surpassing widely adopted statistical models at both the individual and population levels, while providing foundational evidence for integrating CAVA into existing VA systems. These findings emphasize the potential of CAVA to complement physician reasoning and enhance COD estimation, ultimately supporting more accurate and evidence-based health policies and interventions.

## Supplementary Information

Additional file 1. (.pdf) titled ”Central Medical Evaluation Agreement 10 (CMEA-10) codes” with description” ICD-10 code ranges considered in physician agreement” was used to supplement this study.

## Figures and Tables

**Fig. 9:**
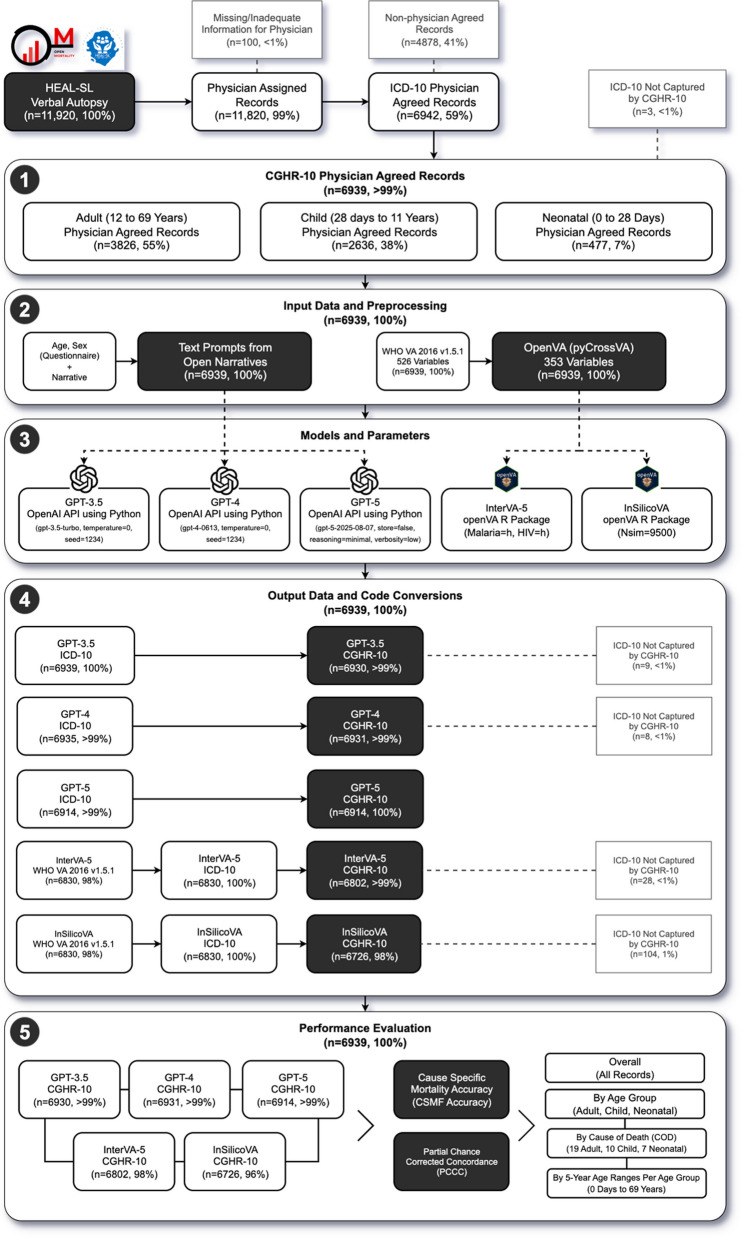
Detailed flow diagram for verbal autopsy coding comparison of 6939 sample deaths in Sierra Leone. This supplements the diagram in Fig. 1, providing additional details on removed records, model specifications, and sub-processes

**Fig. 10:**
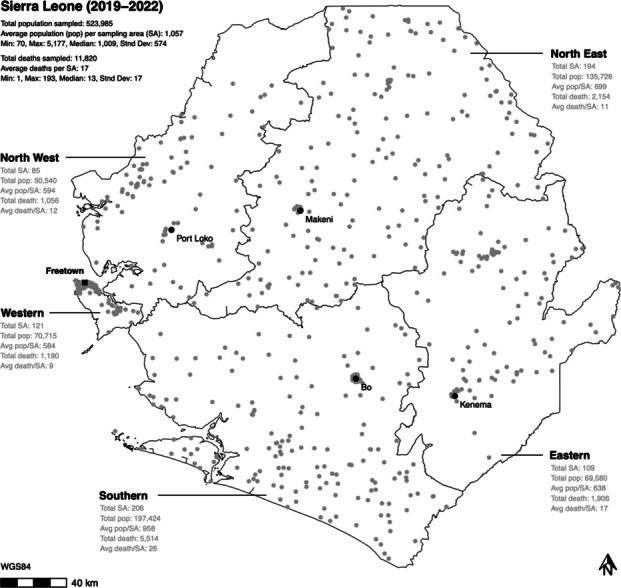
Healthy Sierra Leone (HEAL-SL) verbal autopsy data sampling areas for Sierra Leone from 2019–2022. The boundaries represent Sierra Leone provinces. Each grey point represents the centroid of sampling areas. Pop = population, SA = sampling area

## Data Availability

The datasets generated and/or analysed during the current study are available in the Open Mortality repository, https://openmortality.org, on reasonable request. All code files used and/or analysed during the current study are available in the Github repository, https://github.com/cghr-toronto/wen-et-al-2025-cava.

## Appendix 1

### CGHR-10 Codes

**Table 2:** CGHR-10 codes for adults (12–69 years)

Adult CGHR-10 Code	ICD-10 Range
Acute respiratory infections	H65-H68, H70-H71, J00-J22, J32, J36, J85-J86, P23
Tuberculosis	A15-A16, B90, J65
Diarrhoeal	A00-A09
Unspecified infections	A17-A33, A35-A99, B00-B17, B19-B49, B55-B89, B91-B99, C46, D64, D84, G00-G09, H10, H60, I30, I32-I33, K02, K04-K05, K61, K65, K67, K81, L00-L04, L08, M00-M01, M60, M86, N10, N30, N34, N41, N49, N61, N70-N74, P35-P39, R50, R75, ZZ21
Malaria	B50-B54
Maternal conditions	A34, F53, O00-O08, O10-O16, O20-O99
Nutritional deficiencies	D50-D53, E00-E02, E40-E46, E50-E64, X53-X54
Chronic respiratory	J30-J31, J33-J35, J37-J64, J66-J84, J90-J99, R04-R06, R84, R91
Cancers	C00-C26, C30-C45, C47-C58, C60-C97, D00-D48, D91, N60, N62-N64, N87, R59
Ischemic heart	I20-I25, R55
Stroke	G45-G46, G81-G83, I60-I69
Diabetes mellitus	E10-E14
Other cardiovascular	I00-I03, I05-I15, I26-I28, I31, I34-I52, I70-I99, R00-R01, R03, ZZ23
Liver and alcohol related	B18, F10, K70-K77, R16-R18, X45, Y15, Y90-91
Other noncommunicable	D55-D63, D65-D83, D86, D89, E03-E07, E15-E35, E65-E68, E70E90, F00-F09, F11-F52, F54-F99, G10-G37, G40-G41, G43-G44, G50-G80, G84-G99, H00-H06, H11-H59, H61-H62, H69, H72-H95, K00-K01, K03, K06-K14, K20-K31, K35-K38, K40-K60, K62-K64, K66, K78-K80, K82-K93, L05, L10-L99, M02-M54, M61-M85, M87-M99, N00-N08, N11-N29, N31-N33, N35-N40, N42-N48, N50N59, N75-N86, N88-N99, Q00-Q99, R10-R15, R19-R23, R26-R27, R29-R49, R56, R63, R70-R74, R76-R77, R80-R82, R85-R87, R90, ZZ25
Road and transport injuries	V01-V99, Y85
Suicide	X60-X84
Other injuries	S00-S99, T00-T99, W00-W99, X00-X44, X46-X52, X55-X59, X85-X99, Y00-Y14, Y16-Y84, Y86, Y89, Y92-Y98, ZZ27
Ill-defined	R02, R07-R09, R25, R51-R54, R57-R58, R60-R62, R64-R69, R78-R79, R83, R89, R92-R94, R96, R98-R99

**Table 3:** CGHR-10 codes for children and neonates

Child CGHR-10 Code (28 days to 11 years)	ICD-10 Range
Pneumonia	A37, H65-H68, H70-H71, J00-J22, J32, J36, J85-J86, P23, U04
Diarrhoeal	A00-A09
Malaria	B50-B54
Other infections	A15-A28, A30-A36, A38-A44, A46, A48-A71, A74-A75, A77-A99, B00B09, B15-B27, B30, B33-B49, B55-B60, B64-B83, B85-B92, B94-B97, B99, G00-G09, H10, H60, I30, I32-I33, I39-I41, J65, K02, K04-K05, K61, K65, K67, K81, L00-L04, L08, M00-M01, M60, M86, N10, N30, N34, N41, N49, N61, N70-N74, P35-P39, R50, R75, U00, Y95, ZZ11
Congenital anomalies	P01, P05, P07, P21, Q00-Q99
Epilepsy, leukaemia, and other noncommunicable	C00-C97, D01-D48, D55-D89, E03-E35, E65-E90, F00-F02, F73, G10G99, H00-H06, H11-H59, H61-H62, H69, H72-H95, I00-I28, I31, I34-I38, I42-I99, J30-J31, J33-J35, J37-J47, J60, J64, J66-J70, J80-J82, J84, J90-J99, K00-K01, K03, K06-K60, K62-K63, K70-K80, K82-K93, L05, L10-L99, M02-M54, M61-M85, M87-M99, N00-N08, N11-N29, N31N33, N35-N40, N42-N48, N50-N51, N60, N62-N64, N75-N99, P04, P08, P27, P51, P53-P60, P70-P72, P74-P76, P78, P80-P83, P92-P94, R00-R01, R03-R06, R11-R23, R26-R27, R29-R49, R55-R56, R59, R63, R70-R74, R76-R77, R80-R82, R84-R87, R90-R91, ZZ12-ZZ13, ZZ15
Injuries	S00-S99, T00-T98, V01-V99, W00-W99, X00-X52, X57-X99, Y00-Y91, Y97-Y98
Nutritional deficiencies	D50-D53, E00-E02, E40-E46, E50-E56, E59-E61, E63-E64, X53-X54
Other	D00, F03-F72, F74-F99, P00, P02-P03, P10-P15, P20, P22, P24-P26, P28-P29, P50, P52, P61, P77, P90-P91
Ill-defined	P96, R02, R07, R09-R10, R25, R51-R54, R57-R58, R60-R62, R64, R68-R69, R78-R79, R83, R89, R92-R99
Neonate CGHR-10 Code (< 28 days)	
Prematurity & low birthweight	D64, O60, P01, P05, P07, P22, P25-P28, P52, P61, P77, P80, P92, R04
Neonatal infections	A00-A09, A20-A28, A32-A35, A37-A44, A46, A48-A49, A68A70, A74-A75, A77-A79, A81-A90, B54, B95-B96, G00-G09, H10, H60, H65-H68, H70-H71, I30, I32-I33, I39-I41, J00-J22, J32, J36, J85-J86, K65, K67, K81, L00-L04, L08, M00-M01, M60, M86, N10, N30, N34, N41, N49, N61, O85, P23, P35,P39, P58-P59, P63, U04
Birth asphyxia and birth trauma	G40, P00, P02-P03, P10-P15, P20-P21, P24, P29, P50-P51, P90-P91, R06, W79, Z37
Stillbirth	P95
Congenital anomalies	C76, Q00-Q99
Other	A15-A19, A30-A31, A36, A50-A67, A71, A80, A91-A99, B00-B09, B15-B27, B30, B33-B53, B55-B60, B64-B83, B85-B92, B94, B97, B99, C00-C75, C77-C97, D00-D48, D50-D53, D55-D63, D65-D89, E00-E35, E40-E46, E50-E56, E59-E61, E63-E90, F00-F99, G10-G39, G41-G99, H00-H06, H11-H59, H61-H62, H69, H72-H95, I00-I28, I31, I34-I38, I42-I99, J30, J31, J33-J35, J37-J47, J60, J64-J70, J80-J82, J84, J90J99, K00-K63, K70-K80, K82-K93, L05, L10-L99, M02-M54, M61-M85, M87-M99, N00-N08, N11-N29, N31-N33, N35N40, N42-N48, N50-N51, N60, N62-N64, N70-N99, P04, P08, P53-P57, P60,P70-P72, P74-P76, P78, P81-P83, P93-P94, R00-R01, R03-R05, R11-R23, R26-R27, R29-R36, R39-R50, R55-R56, R59, R63, R70-R77, R80-R82, R84-R87, R90-R91, S00-S99, T00-T98, U00, V01-V99, W00-W78, W80-W99, X00-X54, X57-X99, Y00-Y91, Y95, Y97-Y98
Ill-defined	P96, R02, R07, R09-R10, R25, R51-R54, R57-R58, R60-R62, R64, R68-R69, R78-R79, R83, R89, R92-R99

## Appendix 2

### Details on methods

This section provides additional details on the methods described in Section 2. An overview of the methods used in this study is seen in Figure B1 as a five-step process. Section B.1 provides details on the preprocessed data used for modelling. Section B.2 describes the data and parameter inputs and outputs for each model, while Section B.3 details the evaluation of model outputs at the individual and population level across different CODs, age groups, and ages. Publicly available data for reproducing figures and tables is made available on Harvard Dataverse, see [106].

### CGHR-10 physician-agreed records

Initially, 11,920 records were collected from dual-coded EVA in the HEAL-SL study. Physicians were able to assign CODs for 11,820 of the 11,920 records, where 100 of these records could not be assigned a COD due to missing or inadequate information (e.g., low quality narrative, data loss). The 11,820 physician coded records were further filtered for records where both physicians agreed on the assigned codes (records that were not reconciled or adjudicated) resulting in 6942 physician agreed records (based on comparisons using CMEA-10 codes, see Additional File 1). The 6942 records were converted into CGHR-10 codes (see Appendix A) that generalized ICD-10 codes into 19, 10, and 7 categories for the adult (12 to 69 years), child (28 days to 11 years), and neonatal (under 28 days) age groups. After conversion, a final total of 6939 physician agreed records (3826 adult, 2636 child, and 477 neonatal) were used for modelling and performance evaluation, where three records were removed as their ICD-10 codes did not have a matching CGHR-10 code.

The 6,939 physician agreed records were collected using VA from the HEAL-SL study between 2019–2022, where deaths were collected using nationwide representative sampling units across Sierra Leone (Figure B2). The distribution of the study data are shown by CGHR-10 causes of death in Table B4. All age groups had relatively evenly distributed female and male records (44–55% of 6939 records each). Across CODs, there were noticeably more female records for cancers (65%), and maternal conditions (100%), while more male records for chronic respiratory diseases (61%), other noncommunicable diseases (61%), other injuries (77%), road and transport injuries (71%), and tuberculosis (68%). Most records were coded by physicians as malaria for adults (20%) and children (52%), and stillbirth (36%) and infections (21%) for neonates. Suicide, congenital anomalies, nutritional deficiencies, and other had low sample sizes for each age group (< 1% of total records for each age group). CODs with high physician agreement (> 70%) include malaria (77%), road/transport injuries (75%), and other injuries (72%) for adults, malaria (85%) and injuries (76%) for children, and stillbirth (76%) for neonates. CODs with low physician agreement (< 40%) were cancers (20%), pneumonia (34%), and unspecified infections (37%) for adults, and nutritional deficiencies (39%) and pneumonia (31%) for children. Table B5 shows the distribution of the study data by age. Across ages, there were more male records for 50–59 years (60–62%), while all other records had between 49–59% female and male records. Most records were in the 65–69 years age range for adults (15%), 1–5 years for children (62%), and 0–6 days for neonates (83%). Physician agreement was similar at around 50–70% for all age ranges.

### Modelling details

Each model (GPT-3.5, GPT-4, GPT-5, InSilicoVA, and InterVA-5) required pre-processing of the 6939 records into input data, and standardization of output COD codes from models for performance evaluation as not all models produced comparable codes across outputs. Although each model can assign multiple CODs per record, only the first generated COD response from GPT-3.5/4/5, and the most probable COD from InterVA-5 and InSilicoVA were used for evaluation. Section B.2.1 describes the input data and parameters for each model, while Section B.2.3 details the outputs from running each model.

**Table 4:** Study data by cause of death

Age Group	Cause of Death (COD)	Agree	Female	Male	Total
Adult (*n* = 3826, 55.1%, 18 CODs) - Female (*n* = 1681, 43.9%) - Male (*n* = 2145, 56.1%) - Agree (54.9% of 6970)	Cancers	20.4% of 240	32 (65.3%)	17 (34.7%)	49 (1.3%)
	Chronic Respiratory	57.7% of 130	29 (38.7%)	46 (61.3%)	75 (2%)
	Diabetes Mellitus	65.9% of 41	14 (51.9%)	13 (48.1%)	27 (0.7%)
	Diarrhoeal	50.4% of 407	102 (49.8%)	103 (50.2%)	205 (5.4%)
	Ill-Defined	45.5% of 257	56 (47.9%)	61 (52.1%)	117 (3.1%)
	Ischemic Heart	57.1% of 294	89 (53%)	79 (47%)	168 (4.4%)
	Liver/Alcohol	52.7% of 243	58 (45.3%)	70 (54.7%)	128 (3.3%)
	Malaria	77% of 1038	372 (46.6%)	427 (53.4%)	799 (20.9%)
	Maternal	53.5% of 243	130 (100%)	N/A	130 (3.4%)
	Other Cardiovascular	41.5% of 258	59 (55.1%)	48 (44.9%)	107 (2.8%)
	Other Noncommunicable	49.2% of 841	160 (38.6%)	254 (61.4%)	414 (10.8%)
	Other Injuries	72.1% of 495	83 (23.2%)	274 (76.8%)	357 (9.3%)
	Pneumonia	34.3% of 306	48 (45.7%)	57 (54.3%)	105 (2.7%)
	Road/Transport Injuries	75.1% of 334	73 (29.1%)	178 (70.9%)	251 (6.6%)
	Stroke	66.5% of 498	147 (44.4%)	184 (55.6%)	331 (8.7%)
	Suicide	50% of 6	N/A	3 (100%)	3 (0.1%)
	Tuberculosis	62% of 276	54 (31.6%)	117 (68.4%)	171 (4.5%)
	Unspecified Infections	36.6% of 1062	175 (45%)	214 (55%)	389 (10.2%)
Child (*n* = 2636, 38%, 9 CODs) - Female (*n* = 1290, 48.9%) - Male (*n* = 1346, 51.1%) - Agree (65.6% of 4016)	Congenital Anomalies	6.7% of 15	1 (100%)	N/A	1 (0%)
	Diarrhoeal	55.6% of 315	79 (45.1%)	96 (54.9%)	175 (6.6%)
	Epilepsy, Leuk. & Other Noncom	43.5% of 262	61 (53.5%)	53 (46.5%)	114 (4.3%)
	Ill-Defined	57.4% of 122	34 (48.6%)	36 (51.4%)	70 (2.7%)
	Injuries	75.8% of 178	51 (37.8%)	84 (62.2%)	135 (5.1%)
	Malaria	84.6% of 1634	680 (49.2%)	702 (50.8%)	1382 (52.4%)
	Nutritional Deficiencies	39.3% of 28	7 (63.6%)	4 (36.4%)	11 (0.4%)
	Other Infections	55.5% of 1201	338 (50.7%)	329 (49.3%)	667 (25.3%)
	Pneumonia	31% of 261	39 (48.1%)	42 (51.9%)	81 (3.1%)
Neonate (*n* = 477, 6.9%, 7 CODs) - Female (*n* = 227, 47.6%) - Male (*n* = 250, 52.4%) - Agree (58.7% of 813)	Birth Asphyxia And Trauma	55.7% of 185	38 (36.9%)	65 (63.1%)	103 (21.6%)
	Congenital Anomalies	28.6% of 7	2 (100%)	N/A	2 (0.4%)
	Ill-Defined	52.3% of 44	11 (47.8%)	12 (52.2%)	23 (4.8%)
	Infections	52.7% of 188	49 (49.5%)	50 (50.5%)	99 (20.8%)
	Other	29.4% of 17	2 (40%)	3 (60%)	5 (1%)
	Prematurity And Low Birthweight	50.3% of 145	39 (53.4%)	34 (46.6%)	73 (15.3%)
	Stillbirth	75.8% of 227	86 (50%)	86 (50%)	172 (36.1%)

^*^Agreement refers to records where two physicians agree on the cause of death based on CMEA-10 code equivalency. This represents a proportion of all 11,799 physician-coded records, regardless of agreement (e.g., 55.1% of all 6970 adult physician-coded records agree). All other numbers refer to the 6939 subset of records with agreement only

**Table 5:** Study data by age range

Age Group	Age Range	Agree^*^	Female	Male	Total
Adult (*n* = 3826, 55.1%, 18 CODs) - Female (*n* = 1681, 43.9%) - Male (*n* = 2145, 56.1%) - Agree (54.9% of 6970)^*^	12–14 Years	64.3% of 210	51 (37.8%)	84 (62.2%)	135 (3.5%)
	15–19 Years	59.6% of 451	115 (42.8%)	154 (57.2%)	269 (7%)
	20–24 Years	56% of 491	146 (53.1%)	129 (46.9%)	275 (7.2%)
	25–29 Years	59.2% of 595	159 (45.2%)	193 (54.8%)	352 (9.2%)
	30–34 Years	54.6% of 626	174 (50.9%)	168 (49.1%)	342 (8.9%)
	35–39 Years	54.8% of 615	153 (45.4%)	184 (54.6%)	337 (8.8%)
	40–44 Years	51.9% of 615	134 (42%)	185 (58%)	319 (8.3%)
	45–49 Years	52.9% of 595	148 (47%)	167 (53%)	315 (8.2%)
	50–54 Years	52.2% of 647	134 (39.6%)	204 (60.4%)	338 (8.8%)
	55–59 Years	52.8% of 483	96 (37.6%)	159 (62.4%)	255 (6.7%)
	60–64 Years	51.2% of 613	128 (40.8%)	186 (59.2%)	314 (8.2%)
	65–69 Years	55.9% of 1029	243 (42.3%)	332 (57.7%)	575 (15%)
Child (*n* = 2636, 38%, 9 CODs) - Female (*n* = 1290, 48.9%) - Male (*n* = 1346, 51.1%) - Agree (65.6% of 4016)^*^	1–5 Months	58.1% of 530	146 (47.4%)	162 (52.6%)	308 (11.7%)
	6–11 Months	64.4% of 489	160 (50.8%)	155 (49.2%)	315 (11.9%)
	1–5 Years	68.6% of 2382	822 (50.3%)	811 (49.7%)	1633 (61.9%)
	6–11 Years	61.8% of 615	162 (42.6%)	218 (57.4%)	380 (14.4%)
Neonate (*n* = 477, 6.9%, 7 CODs) - Female (*n* = 227, 47.6%) - Male (*n* = 250, 52.4%) - Agree (58.7% of 813)^*^	0–6 Days	60.6% of 652	184 (46.6%)	211 (53.4%)	395 (82.8%)
	7–27 Days	50.9% of 161	43 (52.4%)	39 (47.6%)	82 (17.2%)

^*^Agreement refers to records where two physicians agree on the cause of death based on CMEA-10 code equivalency. This represents a proportion of all 11,799 physician-coded records, regardless of agreement (e.g., 55.1% of all 6970 adult physician-coded records agree). All other numbers refer to the 6939 subset of records with agreement only

#### Input data and preprocessing

For GPT-3.5, GPT-4, and GPT-5, 6939 text prompts were generated for each physician agreed record as input to instruct the models to assign CODs based on the open narratives. Two types of text prompts were used: user prompts and system prompts. System prompts contained textual instructions to assign the role of a physician ICD-10 coder with expertise in Sierra Leone. The following system prompt was used: *You are a physician with expertise in determining underlying causes of death in Sierra Leone by assigning the most probable ICD−10 code for each death using verbal autopsy narratives. Return only the ICD−10 code without description. E. g. A00. If there are multiple ICD−10 codes, show one code per line.*

User prompts contained textual instructions to perform coding of VA records based on the age, sex, and narrative of the deceased. The following template was used to generate user prompts for each record, where < age > and < sex > from the questionnaire, and < narrative > from the narratives, were replaced with values from the data: *Determine the underlying cause of death and provide the most probable ICD−10 code for a verbal autopsy narrative of a* <*age*> *years old* <*sex*> *death in Sierra Leone:* <*narrative*>

We instruct GPT models to provide ICD-10 codes, as it mirrors physician practice and has been widely adopted and well-studied, enabling standardized comparison across different analyses [107, 108]. In addition, ICD-10 codes are granular and allow for the flexibility of converting to CGHR-10, WHO VA, or any other codes that are based on ICD-10. Thus, grouping ICD-10 codes enables stricter and more standardized definitions of COD categories, ensuring that comparable categories across different coding systems can be accurately aligned, and that observed results are not influenced by variations in coding systems or category definitions [109, 110]. Note that it was not not necessary to provide ICD-10 codes to GPT models as they already had knowledge of them. For InterVA-5 and InSilicoVA, the standardized questionnaire data from the HEAL-SL EVA were first converted into 2016 World Health Organization (WHO) VA questionnaire revision 1.5.1 Open Data Kit (ODK) format [111, 112] consisting of 526 variables [113], followed by further conversion into OpenVA format [42] consisting of 353 variables [114] using the pyCrossVA version 0.97 Python package [115]. The 6939 records were all converted into OpenVA formatted records for InterVA-5 and InSilicoVA.

#### Models and parameters

The GPT-3.5, GPT-4, and GPT-5 Application Programming Interfaces (APIs) were accessed using Python version 3.11.7 and used to assign CODs for each record. We configure stateless calls (starting new sessions per call without any memory of previous sessions) to OpenAI’s API, where GPT-3.5 and GPT-4 used the Chat Completions API [116] (stateless by default) and GPT-5 used the Responses API [117] (requires parameter *store* set to *false* for statelessness). Stateless calls ensure that previous records and their order did not influence COD assignment for future records, potentially reducing output randomness. GPT-3.5 used the *gpt-3.5-turbo* model, GPT-4 used the *gpt-4–0613* model, and GPT-5 used the *gpt-2025–08-07* model. The parameter *temperature* for GPT-3.5 and GPT-4, representing the sampling temperature ranging from 0 to 2 (default of 1), was set to 0 to produce more deterministic outputs [118]. Higher values closer to 2 may produce less deterministic outputs, while lower values closer to 0 produce more deterministic outputs. Another parameter for GPT-3.5/4, *seed* was fixed at a constant value of 1234 to minimize randomness in token generation and improve output consistency [73]. Despite this setting, we note that GPT-3.5/4 were non-deterministic models, where using a constant seed value increases the likelihood of identical outputs across runs, but does not guarantee them. For GPT-5, the *temperature* and *seed* parameters were no longer available from the API, and we set the parameter *reasoning* to *minimal* and parameter *verbosity* to *low* as an alternative for producing more deterministic outputs [71, 72]. The *reasoning* parameter controls for the number of reasoning tokens, affecting the ability of GPT-5 to outline steps of thinking for refining the output. Values available were *minimal*, *low*, *medium*, or *high*, with higher values increasing the number of reasoning tokens to better refine outputs. We note that we use low reasoning for GPT-5 to reduce the likelihood of inconsistent outputs as the generation of more reasoning tokens increases output randomness. The *verbosity* parameter (nested under the *text* parameter) controls the number of output tokens, adjusting GPT-5 to provide more concise or more detailed output. Values available were *high*, *medium, or low*, where lower verbosity produces more concise output and higher verbosity produces more detailed output. We use low verbosity for GPT-5 to produce more concise and deterministic outputs. The *store* parameter was also to *false* for stateless GPT-5 sessions using the Responses API [119]. The *openVA* R package was used to run InterVA-5 and InSilicoVA models to assign CODs for each record in R version 4.3.1. The *openVA* package version 1.1.1 used dependent packages InterVA5 version 1.1.3 and InSilicoVA version 1.4.0. The *Nsim* (number of iterations to run) parameter [120] for InSilicoVA was set to 9500, while the HIV (level of prevalence of human immunodeficiency virus) and Malaria (level of prevalence of Malaria) parameters [121] for InterVA-5 were both set to *h* (high) reflecting HIV and Malaria disease assumptions in Sierra Leone [122, 123]. Note that the default value of *Nsim* = *10000* for InSilicoVA ran until 9500 iterations before it stopped due to errors, thus *Nsim* = *9500* was used and ran successfully.

#### Output data and code conversion

Of the 6939 input records, GPT-3.5, GPT-4, GPT-5, InterVA-5, and InSilicoVA were able to assign CODs for 6939 (100%), 6935 (> 99%), 6916 (> 99%), 6830 (98%), and 6830 (98%) records respectively. All 6830 (100%) InterVA-5 and InSilicoVA records with WHO VA 2016 v1.5 output codes [57] were converted into ICD-10 codes respectively. After all model outputs were converted to ICD-10 codes, they were further converted to CGHR-10 codes. The 6939 GPT-3.5, 6935 GPT-4, and 6916 GPT-5 output records with ICD-10 codes were converted into 6930 (> 99%), 6931 (> 99%), and 6196 (100%) records with CGHR-10 codes, where < 1% (9, 8, and 0) records did not have matching CGHR-10 codes respectively. The 6830 InterVA-5 and InSilicoVA records with ICD-10 codes were converted into 6802 (> 99%) and 6726 (98%) records with CGHR-10 codes respectively, where 28 (< 1%) and 104 (1%) of records could not be converted into CGHR-10 codes.

### Performance evaluation details

The performance of GPT-3.5, GPT-4, GPT-5, InSilicoVA, and InterVA-5 models were evaluated with metrics at the population and individual-level by comparing their CGHR-10 COD outputs for 6939 records to physician COD assignments. Section B.3.1 describes CSMF accuracy in detail for evaluating models on the population level, Section B.3.2 describes PCCC for evaluating models on the individual-level. Records that were assigned a COD by physicians, but not by a model were considered an incorrect COD assignment by the model. CSMF accuracy and PCCC were calculated for each model overall and by three age groups (adult, child, and neonatal), then further into age and COD for each age group.

#### Cause Specific Mortality Fraction (CSMF) accuracy

CSMF accuracy measures the performance of models at the population level, comparing distributions of CODs between the physicians and the models [58]. To calculate CSMF accuracy, ${\mathrm{CSM}}{\mathrm{F}}_{\mathrm{j}}$ was calculated as is the fraction of physician or model records for cause *j*, given by dividing the number of records for cause *j* with the total number of records as seen in Equation B1. Then, the *CSMFMaximumError*, representing the worst possible model, is calculated using Equation B2. Finally, the CSMF accuracy is given by Equation B3, where *k* is the number of causes, *j* is a cause, $CSM{F}_{j}^{true}$ is the true physician CSMF for cause *j*, and $CSM{F}_{j}^{pred}$ is the prediction model CSMF for cause *j*. CSMF accuracy ranges from 0 to 1, where 1 means that the model completely matched the physician COD distribution and 0 means that it did not match the distribution.

B1 $${CSM}{F}_{j}=\frac{{Record}{s}_{j}}{{Records}}$$

B2 $$CSMFMaximumError = 2(1 - Min(CSM{F}_{j}^{true})$$

B3 $$CSMF\;Accuracy=1-\frac{\sum_{j=1}^k\left|CSMF_j^{true}-CSMF_j^{pred}\right|}{CSMF\;MaximumError}$$

#### Partial Chance Corrected Concordance (PCCC)

PCCC measures the performance of models at the individual-level, comparing COD assignments between the physicians and models on a record by record basis, correcting for COD assignments made purely by chance [58]. PCCC is given by Equation B5, where *k* is the number of top COD assignments from the model to consider, *N* is number of causes, and *C* is fraction of records where the physician COD assignment is one of the top COD assignments from the model. For this study, *k* was set to 1, making *C* equivalent to the fraction of true positives *TP* or records, where the physician COD assignment is equal to the model COD assignment as shown in Equation B4. Higher PCCC values closer to 1 indicate that model COD assignments are similar to physician COD assignments, while values closer to 0 indicate that they are not similar to physicians.

B4 $$C=\frac{TP}{Records}$$

B5 $$PCCC\left(k\right)=\frac{C-\frac kN}{1-\frac kN}$$

## Appendix 3

### Experiment on repeated runs of GPT models

A short experiment was conducted to test the consistency of GPT-3.5, GPT-4, and GPT-5 outputs repeated on the same record. 100 records, sampled randomly with approximately equal proportions across age groups, CODs, and survey rounds 1 and 2, were used to test repeated runs of each GPT model. Each record from the 100 records was rerun 10 times through each GPT model, resulting in ten COD outputs per record. The ICD-10 codes were then converted to CGHR-10 codes and tested for consistency, where completely inconsistent results had different ICD-10 or CGHR-10 codes for each of the 10 reruns (1 times +), and completely consistent results had an identical ICD-10 or CGHR-10 code for all 10 reruns (10 times), on identical records. The results are shown in Table C6. For all 100 records, GPT-4 assigned an identical ICD-10 and CGHR-10 code for identical records 5 times or more out of 10, while GPT-3.5 and GPT-5 had similar outputs, except that GPT-3.5 and GPT-5 had 99 records for CGHR-10 codes and ICD-10 codes respectively. GPT-3.5 and GPT-4 were the most consistent, assigning identical ICD-10 codes over reruns for more than 70 records (71 for GPT-3.5 and 75 for GPT-4), and identical CGHR-10 codes for more than 80 records (82 for GPT-3.5 and 84 for GPT-4) each. These numbers steadily increase (at 1 to 9 records) to over 90 records with a reduction in the number of times that an identical code was assigned (e.g., from 9 times + of 10 to 6 times + of 10). In contrast, GPT-5 was inconsistent with only 30 and 59 records with identical ICD-10 and CGHR-10 code assignments over reruns respectively. A steeper increase of 5 to 15 records was observed with a reduction in the number of times that an identical code was assigned. We note that the temperature and seed parameters that were available in GPT-3.5/4, and unavailable in GPT-5, were suspect for these differences. Thus, GPT models do not always guarantee identical outputs when repeated on the same record (10 times out of 10), despite parameter adjustments for more deterministic outputs (e.g., temperature set to 0, seed set to a constant of 1234, verbosity set to low, reasoning set to minimal). This experiment revealed that GPT-3.5/4 did produce identical outputs when rerun for more than half the records, while this was true only for CGHR-10 codes in the case of GPT-5. For most records (approximately 90%), GPT-3.5 and GPT-4 produced the same outputs for identical records 7 + times of 10, while this was observed at 6 + times out of 10 for GPT-5.

**Table 6:** Records with identical GPT-3.5/4/5 outputs based on 10 repeated reruns of 100 records

# of Times with Identical GPT-3.5 Outputs	ICD-10 Records	CGHR-10 Records
1 times + (inconsistent)	100	100
2 times +	100	100
3 times +	100	100
4 times +	100	100
5 times +	100	99
6 times +	95	98
7 times +	90	95
8 times +	86	92
9 times +	80	89
10 times (consistent)	71	82
# of Times with Identical GPT-4 Outputs	ICD-10 Records	CGHR-10 Records
1 times + (inconsistent)	100	100
2 times +	100	100
3 times +	100	100
4 times +	100	100
5 times +	100	100
6 times +	92	94
7 times +	89	93
8 times +	84	89
9 times +	81	87
10 times (consistent)	75	84
# of Times with Identical GPT-5 Outputs	ICD-10 Records	CGHR-10 Records
1 times + (inconsistent)	100	100
2 times +	100	100
3 times +	100	100
4 times +	100	100
5 times +	99	100
6 times +	80	93
7 times +	67	88
8 times +	55	83
9 times +	42	68
10 times (consistent)	30	59

## Abbreviations

CAVA: Computer Assisted Verbal Autopsy
CCVA: Computer Coded Verbal Autopsy
CGHR: Centre for Global Health Research
CMEA: Central Medical Evaluation Agreement
COD: Cause of Death
CSMF: Cause Specific Mortality Fraction
EVA: Electronic Verbal Autopsy
GPT: Generative Pre-trained Transformer
HEAL-SL: Healthy Sierra Leone
ICD: Internation Classification of Diseases
LLM: Large Language Model
PCCC: Partial Chance Corrected Concordance
VA: Verbal Autopsy
WHO: World Health Organization
