# Correction: Computer assisted verbal autopsy: comparing large language models to physicians for assigning causes to 6939 deaths in Sierra Leone from 2019–2022

**DOI:** 10.1186/s12916-026-04702-5

**Published:** 2026-02-14

**Authors:** Richard Wen, Anteneh Tesfaye Assalif, Andy Sze-Heng Lee, Rajeev Kamadod, Asha Behdinan, Ronald Carshon-Marsh, Catherine Meh, Thomas Kai Sze Ng, Patrick Brown, Prabhat Jha, Rashid Ansumana

**Affiliations:** 1 Centre for Global Health Research, St. Michael’s Hospital, Unity Health Toronto and University of Toronto, 30 Bond St, Toronto, ON M5B 1W8 Canada; 2 College of Medical Sciences, Njala University, Bo, Sierra Leone

**Correction: BMC Med 24, 49 (2025)**

**https://doi.org/10.1186/s12916-025-04584-z**

The original article [1], displays an error in the following sentence: “A majority of CODs had at least one high performing model (0.78–0.99.78.99 for 21 of 30 CODs).”

The text ‘.78.99’ is erroneous and should be disregarded, leaving the sentence read instead as follows: “A majority of CODs had at least one high performing model (0.78–0.99 for 21 of 30 CODs).”
