# Supplementary material for: Computer assisted verbal autopsy: comparing large language models to physicians for assigning causes to 6939 deaths in Sierra Leone from 2019–2022
Source: BMC Med. 2025 Dec 24;24:49. doi: 10.1186/s12916-025-04584-z (PMC12849170; doi:10.1186/s12916-025-04584-z)
Supplement: Supplementary file 1 — Additional file 1. (.pdf) titled ”Central Medical Evaluation Agreement 10 (CMEA-10) codes” with description” ICD-10 code ranges considered in physician agreement” was used to supplement this study. [file 12916_2025_4584_MOESM1_ESM.pdf]

Central Medical Evaluation Agreement 10 (CMEA-10) Code	International Classification of Diseases Revision 10 (ICD-10) Code
Tuberculosis	A15-19,B90,J65
Syphilis	A51-53
Other sexually transmitted infections (excl. HIV/AIDS)	A54-64,N70-74
HIV/AIDS	B20-24,C46,D84,R75
Diarrhoeal diseases	A00-09
Tetanus	A33,A35
Measles	B01,B05
Other vaccine preventable diseases (1)	A36-37,B26
Poliomyelitis	A80,B91
Other vaccine preventable diseases (2)	B03,B06
Meningitis/Encephalitis	A39,A81,A83-89,G00-09
Hepatitis	B15-19
Malaria	B50-54
Protozoal diseases	B55-58,B60,B64
Leprosy	A30,B92
Arthropod-borne viral fevers	A90-96,A98-99
Helminthiases	B65-83
Acute respiratory infections - Upper (1)	H65-68,H70-71
Acute respiratory infections - Upper (2)	J00-06,J32,J36
Acute respiratory infections - Lower	J09-18,J20-22,J85-86,U04
Severe systemic infections	A20-28,A31,U80-81,U88-89
Other infectious diseases (1)	A50,A65-67,A69
Other infectious diseases (2)	B00,B02,B04,B07-09,B25,B27,B30,B33-49
Other infectious diseases (3)	B85-89
Other infectious diseases (4)	P35,P37
Obstetric haemorrhage	O20,O43-46,O67,O71-72
Maternal sepsis	A34,O23,O41,O85-86,O91
Hypertensive disorders of pregnancy	O11-16
Other maternal conditions (1)	O64-66
Abortion or miscarrage	O00-08
Other maternal conditions (2)	F53,O21-22,O24-26,O28-36,O40,O42-43,O47-48,O60-63,O68-70,O73-75,O80-84,O87-90,O92,O94-99
Low birth weight/preterm	P01,P05,P07,P22,P25-29,P52,P61,P77

Birth asphyxia and birth trauma	P02-03,P10-15,P20-21,P24,P50
Other perinatal conditions - (1)	P00,P04
Other perinatal conditions - (2)	P51,P53-60
Other perinatal conditions - (3)	P70-72,P74,P80-81,P83,P92-94,P96
Other perinatal conditions - (4)	P75-76,P78
Protein-energy malnutrition	E40-46,X53-54
Iron, vitamin deficiencies, and nutritional anaemias	D50-53,E00-02,E50-56,E58-61,E63-64
Upper aerodigestive cancer	C00-15,C30-32,D10-11
Colon, rectum, and small intestine cancer	C17-21,D12
Other digestive cancer	C23-26,C48,D00-01,D13,D37
Lung & airway cancer	C33-34,C39,D02,D14
Breast cancer	C50,D05,D24,N60,N62-64
Cervix & Uterus cancer	C53-55,D06,N87
Other female genital cancer	C51-52,C56-58,D25-28,D39
Male genital cancer	C60-63,D29,D40
Urinary cancer	C64-68,D30,D41
Brain and eye cancer	C69-72,D31-33,D42-43
Leukemias, lymphomas, and other haematopoetic malignancies	C81-85,C88,C90-96,D45-47
Other neoplasms (1)	C37-38
Other neoplasms (2)	C40-41
Other neoplasms (3)	C43-44
Other neoplasms (4)	C45-49
Other neoplasms (5)	C73-75
Other neoplasms (6)	C76-80,C97
Other neoplasms (7)	D03-04,D07,D09,D15-23,D34-36,D38,D44,D48
Diabetes mellitus	E10-14
Endocrine and immune disorders (1)	D55-77
Endocrine and immune disorders (2)	D80-84,D86,D89
Endocrine and immune disorders (3)	E03-07
Endocrine and immune disorders (4)	E15-16,E20-32,E34-35,E65-68,E70-80,E83-90
Endocrine and immune disorders (5)	R70-77
Endocrine and immune disorders (6)	R80-82
Epilepsy	G40-41,R56
Other neuropsychiatric disorders - Organic	F00-07,F09

Other neuropsychiatric disorders - Substance Use	F11-19
Other neuropsychiatric disorders - Schizophrenia	F20-25,F28-29
Other neuropsychiatric disorders - Mood	F30-34,F38-39
Other neuropsychiatric disorders - Neurotic	F40-45,F48
Other neuropsychiatric disorders - Behavioural	F50-52,F54-55,F59
Other neuropsychiatric disorders - Personality	F60-66,F68-69
Other neuropsychiatric disorders - Retardation	F70-73,F78-79
Other neuropsychiatric disorders - Psychological	F80-84,F88-89
Other neuropsychiatric disorders - Childhood and adolescence	F90-98
Other neuropsychiatric disorders - nervous system	G10-13,G20-26,G30-32,G35-37,G43-47,G50-64,G70-73,G90-99
Other neuropsychiatric disorders - Cerebral Palsy	G80-83
Other neuropsychiatric disorders - ill-defined	R26-27,R29,R40-49
Skin diseases - Bullous & Papulosquamous	L10-14,L40-45
Skin diseases - Dermatitis	L20-30
Skin diseases - Urticaria	L50-54
Skin diseases - Skin and subcutaneous tissue	L55-60,L62,L64,L66,L68,L70,L72,L74-75,L80-95,L97-99
Skin diseases - Skin and subcutaneous tissue (ill-defined)	R20-23
Musculoskeletal disorders	M00-03,M05-25,M30-36,M40-43,M45-51,M53-54,M60-63,M65-68,M70-73,M75-77,M79-85,M87-96,M99
Oral conditions	K00-01,K03,K06-14
Rheumatic heart diseases	I00-02,I05-09,I38
Hypertensive heart diseases	I10-15
Ischemic heart diseases	I11,I20-25,I44,I46,I70,R55,R96
Cerebrovascular diseases	G45-46,G81-83,I60-69
Other cardiovascular diseases	I26-28,I31,I34-37,I42-43,I45,I47-49,I51-52,I70-74,I77-89,I95-99
Circulatory and respiratory systems - Ill-defined	R00-07,R09
Asthma and Chronic obstructive pulmonary disease	J40-47,J63,J93
Lung dieseases due to external agents	J60-62,J64,J66-70
Other chronic respiratory diseases'	J30-31,J33-35,J37-39,J80-82,J84,J90-92,J94-96,J98-99
Gastro-esophageal	K20-23,K25-31

Digestive system - Ill-defined	R11-19
Lliver and alcohol related diseases	F10,K70-77,X45,Y15,Y90-91
Other digestive diseases - Appnedix	K35-38
Other digestive diseases - Hernia	K40-46
Other digestive diseases - Intestine	K50-52,K55-63
Other digestive diseases	K66,K80,K82-83,K85-87,K90-93
Nephritis and nephrosis	N00-08,N11-19
Urolithiasis	N20-23
Other genitourinary system - Kidney & Ureter	N25-29,N31-33,N35-37,N39,N99
Other genitourinary system - Male genital organs	N40,N42-48,N50-51
Other genitourinary system - Female pelvic organs	N75-77,N80-86,N88-98
Other genitourinary system diseases - Ill-defined	R30-36,R39
Congenital anomalies	Q00-07,Q10-18,Q20-28,Q30-45,Q50-56,Q60-93,Q95-99
Road trafic accidents - (1)	V01-09
Road trafic accidents - (2)	V10-19
Road trafic accidents - (3)	V20-29
Road trafic accidents - (4)	V30-39
Road trafic accidents - (5)	V40-49
Road trafic accidents - (6)	V50-59
Road trafic accidents - (7)	V60-69
Road trafic accidents - (8)	V70-79
Road trafic accidents - (9)	V80-89
Road trafic accidents - (10)	V90-94
Road trafic accidents - (11)	V95-97
Road trafic accidents - (12)	V98-99
Poisonings	X40-44,X46-49
Falls	W00-19
Fires	X00-06,X08-09
Drownings	W65-70,W73-74
Venomous snakes, animals, and plants	W57,W60,X20-29
Other unintestional injuries - Inanimate	W20-46,W49
Other unintestional injuries - Animate	W50-56,W58-59,W64
Other unintestional injuries - Threats to breathing	W75-81,W83-84
Other unintestional injuries - Electric current	W85-94,W99
Other unintestional injuries - Heat	X10-19

Other unintestional injuries - Forces of nature	X30-39
Other unintestional injuries - Overexertion	X50-52,X57
Other unintestional injuries - Accidental exposure	X58-59
Other unintestional injuries - Drugs	Y40-59
Other unintestional injuries - Surgical and Medical care	Y60-66,Y69
Other unintestional injuries - Diagnostic and therapeutic use	Y70-82
Other unintestional injuries - Abnormal reaction	Y83-84
Other unintestional injuries - Sequelae of external causes	Y85-89
Self-inflicted injuries (suicide)	X60-84
Interpersonal violence and other intentional injuries - Assault	X85-99,Y00-09
Interpersonal violence and other intentional injuries - War	Y35-36
Undetermined intent	Y10-14,Y16-34,Y96-98
Other ill-defined and abnormal findings	R00-07,R09,R51-53,R57-69,R78-79,R90-96,R98
